# Performance of unscented Kalman filter tractography in edema: Analysis of the two-tensor model

**DOI:** 10.1016/j.nicl.2017.06.027

**Published:** 2017-06-26

**Authors:** Ruizhi Liao, Lipeng Ning, Zhenrui Chen, Laura Rigolo, Shun Gong, Ofer Pasternak, Alexandra J. Golby, Yogesh Rathi, Lauren J. O’Donnell

**Affiliations:** a Brigham and Women's Hospital, Harvard Medical School, Boston, MA, USA; b Massachusetts Institute of Technology, Cambridge, MA, USA; c Shanghai Changzheng Hospital, Shanghai, China

**Keywords:** White matter, Diffusion MRI, Edema, Tractography, DTI

## Abstract

Diffusion MRI tractography is increasingly used in pre-operative neurosurgical planning to visualize critical fiber tracts. However, a major challenge for conventional tractography, especially in patients with brain tumors, is tracing fiber tracts that are affected by vasogenic edema, which increases water content in the tissue and lowers diffusion anisotropy. One strategy for improving fiber tracking is to use a tractography method that is more sensitive than the traditional single-tensor streamline tractography.

We performed experiments to assess the performance of two-tensor unscented Kalman filter (UKF) tractography in edema. UKF tractography fits a diffusion model to the data during fiber tracking, taking advantage of prior information from the previous step along the fiber. We studied UKF performance in a synthetic diffusion MRI digital phantom with simulated edema and in retrospective data from two neurosurgical patients with edema affecting the arcuate fasciculus and corticospinal tracts. We compared the performance of several tractography methods including traditional streamline, UKF single-tensor, and UKF two-tensor. To provide practical guidance on how the UKF method could be employed, we evaluated the impact of using various seed regions both inside and outside the edematous regions, as well as the impact of parameter settings on the tractography sensitivity. We quantified the sensitivity of different methods by measuring the percentage of the patient-specific fMRI activation that was reached by the tractography.

We expected that diffusion anisotropy threshold parameters, as well as the inclusion of a free water model, would significantly influence the reconstruction of edematous WM fiber tracts, because edema increases water content in the tissue and lowers anisotropy. Contrary to our initial expectations, varying the fractional anisotropy threshold and including a free water model did not affect the UKF two-tensor tractography output appreciably in these two patient datasets. The most effective parameter for increasing tracking sensitivity was the generalized anisotropy (GA) threshold, which increased the length of tracked fibers when reduced to 0.075. In addition, the most effective seeding strategy was seeding in the whole brain or in a large region outside of the edema.

Overall, the main contribution of this study is to provide insight into how UKF tractography can work, using a two-tensor model, to begin to address the challenge of fiber tract reconstruction in edematous regions near brain tumors.

## Highlights

• Reconstruction of edematous white matter from diffusion MRI is investigated.
• The performance of two–tensor unscented Kalman filter (UKF) tractography is assessed.
• The two–tensor model in UKF is analyzed in phantom and patient data experiments.
• Practical guidance on employing the UKF method in neurosurgical patients is provided

## 1 Introduction

A principal goal of modern surgical treatment for brain tumors is to maximize tumor removal while minimizing damage to critical areas of functioning brain (Sanai and Berger, 2008, McGirt et al., 2009). Diffusion magnetic resonance imaging (MRI) is currently the only way to illustrate brain white matter (WM) pathways *in-vivo* and non-invasively (Le Bihan, 2003). Diffusion MRI is able to capture the local microstructure of white matter by measuring the diffusion of particles, usually water molecules. Based on diffusion MRI data, the brain's WM fiber tracts can be virtually reconstructed or traced throughout the brain using computational methods called tractography (e.g. Conturo et al., 1999, Jones et al., 1999, Mori et al., 1999, Weinstein et al., 1999, Basser et al., 2000a, Westin et al., 2002, Lazar et al., 2003, Behrens et al., 2003), a process where fiber trajectories are traced in a stepwise fashion according to local WM models. Diffusion MRI tractography is increasingly used in pre-operative surgical planning to provide precise information about the spatial relationship of tumors to surrounding critical WM tracts, such as the corticospinal tract supporting motor function and the arcuate fasciculus supporting language function (Talos et al., 2003, Nimsky et al., 2005).

Diffusion tensor imaging (DTI), generated by fitting a single-tensor model to the diffusion-weighted MRI (DW-MRI) signals (Basser et al., 1994), is the most commonly used method to model the microstructure of white matter and the current clinical standard. However, the single-tensor model is not able to represent complex WM configurations, such as fiber crossings or partial volume effects (Alexander et al., 2001, Tuch et al., 2002). Due primarily to this limitation, tractography analyses based on DTI underestimate the full anatomical extent of fiber tracts (Kinoshita et al., 2005, Le Bihan et al., 2006, Duffau, 2014, Feigl et al., 2014). Some improvement can be achieved by DTI tractography approaches that employ information from the previous step (Weinstein et al., 1999, Lazar et al., 2003, Westin et al., 2002) during fiber tracking. These approaches improve anatomical accuracy of tractography for neurosurgical planning (Feigl et al., 2014), though they still suffer from the basic limitations of the DTI model.

An additional challenge for tractography for neurosurgical planning is peritumoral edema, where water and protein cross the blood-brain barrier in the region near brain tumors (Jellison et al., 2004, Papadopoulos et al., 2004). The increase in extracellular water changes the diffusion profile, though fiber tracts may still be intact. Tractography methods based on the single tensor DTI model are known to fail in clinically important regions of peritumoral edema (Berman et al., 2004, Pasternak et al., 2009, Nimsky et al., 2016). This is particularly troublesome since the area around the tumor is the most relevant to surgical decision-making.

There is a growing awareness in the neurosurgery community that diffusion models must move beyond the current clinical standard of the diffusion tensor model in order to handle fiber crossings for better anatomical accuracy of fiber tracts (Nimsky, 2014, Farquharson et al., 2013, Fernandez-Miranda, 2013, Kuhnt et al., 2013). While many groups have shown that sophisticated imaging and better mathematical modeling can improve tracing of fiber tracts in the brain (Tuch et al., 2003, Qazi et al., 2009, Descoteaux et al., 2009, Malcolm et al., 2010 Le Bihan and Johansen-Berg, 2012, Rathi et al., 2013), this extensive body of work has been developed for application to neuroscientific studies or in patients without overt brain lesions, where edema is generally not a consideration.

A multi-compartment modeling method provides an alternative to theoretically address the challenges of fiber crossings and edema, but it is faced with the problem of how to get a “best” estimation for the increased number of model parameters. By using a two-compartment tensor model, the effect of edema was reduced on measurements of the tensor trace (Pierpaoli and Jones, 2004), but it is an ill-posed problem to fit the two-tensor model to DW-MRI data. The estimation can be improved by increasing the number of measurements and diffusion weightings, which, however, requires increased acquisition time (Pierpaoli and Jones, 2004). The estimation of a multi-tensor model can also be stabilized by performing it during fiber tracking, and this is the approach that we test here.

In this paper, we show that the challenge of edematous WM fiber reconstruction in clinical data can be addressed to some extent by unscented Kalman filter (UKF) tractography with a two-tensor model (Malcolm et al., 2010), and we analyze the performance of the method. We have recently performed an empirical study demonstrating that two-tensor UKF tractography can trace a higher volume of the arcuate fasciculus (AF) and the corticospinal tract (CST) affected by edema, when compared to single-tensor streamline in neurosurgical patients (Chen et al., 2015, Chen et al., 2016). Here, we investigate the more technical details of the performance of the UKF method in edema, and we assess its performance quantitatively by making reference to individual subject fMRI. The rest of this paper is organized as follows. First, we describe the theory and the model applied, the relevant model parameters, and the synthetic and patient datasets used in this study. Then, we perform experiments to evaluate the performance of UKF tractography in edema, and we investigate its strengths and limitations. Finally, we make recommendations for the use of the presented technique.

## 2 Materials and methods

### 2.1 Theory

#### 2.1.1 Background theory: two-tensor model

A single full diffusion tensor model is commonly used for relating the DW-MRI signals to the structure of white matter (Basser et al. (1994)). For this single-tensor model, the amount of signal loss *S*_*q*_, caused by water diffusion, is modeled by the following equation:

(1) $$S_{q}/S_{0}=e^{-bg_{q}^{T}Dg_{q}}$$

where *S*_0_ is the original signal without diffusion weighting (Basser, 1995); ***g***_***q***_ is the unit direction of a diffusion gradient; ***D*** is the diffusion tensor to be fit; and the factor b is used to describe the gradient timing and strength (Le Bihan et al., 1986).

However, fitting a single tensor to the DW-MRI signals may lead to error, especially when the structure of white matter in a voxel is complicated. Two-tensor models have been proposed for addressing fiber crossing (Tuch et al., 2002, Peled et al., 2006, Pasternak et al., 2008, Qazi et al., 2009) and for removing cerebrospinal fluid (CSF) contamination (Pierpaoli and Jones, 2004, Pasternak et al., 2009).

For the two-tensor model, the signal loss *S*_*q*_ compared to the original signal *S*_0_ can be described by:

(2) $$S_{q}/S_{0}=(1-f)e^{-bg_{q}^{T}D_{1}g_{q}}+fe^{-bg_{q}^{T}D_{2}g_{q}}$$

where *f*, (1 − *f*) are the relative volume fractions of the two compartments, and ***D***_**1**_, ***D***_**2**_ represent the two tensors. In most cases, the two-tensor model has some additional and physically reasonable constraints. Some authors have set the three eigenvalues (*λ*_1_, *λ*_2_, *λ*_2_) to constants (Tuch et al., 2002). The approach followed by many groups (Parker and Alexander, 2005, Peled et al., 2006, Kaden et al., 2007, Friman et al., 2006, Pasternak et al., 2008), including the current implementation of UKF tractography (Malcolm et al., 2010), is to model the smaller two eigenvalues *λ*_2_ and *λ*_3_ as equal, i.e. each tensor is cylindrical.

#### 2.1.2 Background theory: UKF tractography

Streamline tractography starts from an initial seed point and repeatedly propagates the fibers (Conturo et al., 1999, Jones et al., 1999, Mori et al., 1999, Weinstein et al., 1999, Basser et al., 2000a, Westin et al., 2002, Lazar et al., 2003). The propagation follows the equation:

(3) $$\frac{dr(s)}{ds}=t(s)$$

where ***r***(*s*) is a 3D space curve to represent the fiber tract trajectory, and ***t***(*s*) is the local tangent orientation of the curve at *s* (Basser et al. (2000b)). In the fiber propagation, one of the problems is how to fit the model to the DW-MRI signals consistently in each step. In most tractography methods, the model estimations in each voxel are independent and are performed prior to tractography.

In the UKF tractography framework (Malcolm et al., 2010), simultaneous model parameter estimation and tractography are performed using an unscented Kalman filter (Wan and Van Der Merwe, 2000). The model parameters, a ten-component vector representing the two cylindrical tensors, comprise the system state vector ***x***_***s***_ at location *s*, calculated as:

(4) $$x_{s}={\bar{x}}_{s|s-1}+K_{s}(y_{s}-{\bar{y}}_{s|s-1})$$

where ${\bar{x}}_{s|s-1}$ and ${\bar{y}}_{s|s-1}$ are the mean of the sampled states and measurements at *s* predicted from the previously estimated distributions at *s* − 1, ***y***_***s***_ is the DW-MRI signal (current measurement), and ***K***_***s***_ is called Kalman gain, to tune the certainty of the current estimate ***x***_***s***_ and the measurement ***y***_***s***_.

At each point along the traced fiber, the model parameters with their mean and covariance are estimated by the unscented Kalman filter. Then the fiber is propagated forward in the direction of the first tensor's principal eigenvector. This approach offers a causal estimation of the local structure at each step.

The UKF tractography method initializes tracking with two equal tensors at the seedpoint location. As tracking moves away from the initial seedpoint, in the course of fitting the data, the model tends to separate into two different tensors that can represent the tract being traced, plus any other crossing fibers.

### 2.2 Parameters of UKF tractography

The default parameters of UKF were intended for healthy or relatively structurally sound subject data for use in neuroscientific studies. In such studies, false positive connections are avoided when possible. In contrast, in neurosurgical planning it can be useful to increase the number of fibers that can be traced in order to track through difficult regions such as peritumoral edema. We note that the default parameters of UKF tractography work reasonably well in edema, and we have shown that the model performs significantly better than the single-tensor model in a small study of our patient data (Chen et al., 2015, Chen et al., 2016). However, we would like to better understand the behavior of the method in a neurosurgical context. Several parameters of the UKF model are relevant for the application of tracking through edema (Table 1). These parameters were expected to significantly influence the reconstruction of edematous WM fiber tracts, because edema increases water content in the tissue and lowers diffusion anisotropy.

**Table 1:** Parameters of UKF tractography that are relevant to tracking through edema, as well as the values we tested in the patient data. We note that this is not an exhaustive list of all possible parameters, but rather indicates those on which we focused our experiments due to their potential effects on the output. While we did not vary the number of seeds initiated per voxel, the parameter is relevant for increasing the output number of fibers, especially in datasets with larger voxel sizes.

UKF parameter	Measured from	Description	Default	Tested
min FA	tensor 1	Stopping anisotropy threshold	0.15	0.1, 0.15
min GA	DWI data	Stopping anisotropy threshold	0.1	0.05, 0.075, 0.1
free water	multitensor model	Isotropic free water component	Off	On/Off
*q*_*L*_	(input value)	Eigenvalue rate of change	50	100, 200
seeds per voxel	(input value)	Fibers initiated per voxel	1	1

#### 2.2.1 Fractional Anisotropy (FA)

The FA is a normalized variance of the diffusion tensor's eigenvalues (Pierpaoli et al., 1996) and is the traditional stopping threshold for standard single-tensor tractography, and it is frequently lowered to allow tracking to continue farther into edematous regions (Akai et al., 2005). In UKF, the FA that is used as a stopping criterion (min FA) is the FA of the tensor that is being tracked (tensor one):

(5) $$FA=\sqrt{\frac{1}{2}}\sqrt{\frac{{\left(\lambda_{1}-\lambda_{2}\right)}^{2}+{\left(\lambda_{1}-\lambda_{3}\right)}^{2}+{\left(\lambda_{2}-\lambda_{3}\right)}^{2}}{{\lambda_{1}}^{2}+{\lambda_{2}}^{2}+{\lambda_{3}}^{2}}}=\sqrt{\frac{1}{2}}\sqrt{\frac{2{\left(\lambda_{1}-\lambda_{2}\right)}^{2}}{{\lambda_{1}}^{2}+2{\lambda_{2}}^{2}}}$$

where *λ*_1_, *λ*_2_ and *λ*_3_ are the three eigenvalues of tensor one, and *λ*_2_, *λ*_3_ are constrained to be equal in the UKF model.

#### 2.2.2 Generalized Anisotropy (GA)

The GA is a normalized variance of the diffusivities computed from all of the DW-MRIs. This measure does not use any model, tensor or otherwise. GA is computed as:

(6) $$GA=\sqrt{\frac{n}{n-1}}\sqrt{\frac{\sum_{i=1}^{n}{\left(S_{i}-\bar{S}\right)}^{2}}{\sum_{i=1}^{n}{S_{i}}^{2}}}$$

where *S*_*i*_ is the signal measured after the application of diffusion-sensitizing gradient *i*, *n* is the number of the gradients and $\bar{S}$ is the average of the sensitized signals.

#### 2.2.3 free water

The free water parameter controls the inclusion of a free-water model as part of the model used for fitting the DW-MRI signals. The free-water model is a single-tensor model whose three eigenvalues are all equal to 0.003 *mm*^2^/*s* (Pasternak et al., 2009). With the inclusion of the free water term, the multi-tensor model becomes:

(7) $$S_{q}/S_{0}=(1-\omega )\left((1-f)e^{-bg_{q}^{T}D_{1}g_{q}}+fe^{-bg_{q}^{T}D_{2}g_{q}}\right)+\omega e^{-bd}$$

where *ω* is the relative volume fraction of the free-water compartment, *d* is the diffusivity value of the free-water model which is 0.003 *mm*^2^/*s* and the other parameters are the same as those in Eq. (2).

#### 2.2.4 *q*_*L*_

*q*_*L*_ is the expected rate of change of the eigenvalues (system noise). We studied *q*_*L*_ as edema is expected to change the eigenvalues instead of the eigenvectors, which represent the orientation of the fibers. (We note that in the original work (Malcolm et al., 2010), *q*_*L*_ was called *q*_*λ*_.) In UKF tractography, *q*_*L*_ forms part of the injected covariance matrix ***Q*** containing noise bias to eigenvalues and eigenvectors (Malcolm et al., 2010). The measurement noise denoted by ***R***, which relates to the expected noise in the data, was held constant in our experiments (Malcolm et al., 2010).

In UKF tractography, the Kalman gain ***K***_***s***_ is obtained by ***P***_***xy***_***P***_***yy***_^− 1^, where ***P***_***xy***_ is the estimated cross correlation matrix between the state vector ***x***_***s***_ and the DW-MRIs ***y***_***s***_, and ***P***_***yy***_^− 1^ is the estimated covariance matrix of ***y***_***s***_ (Malcolm et al., 2010). Therefore, the increase of *q*_*L*_ will lead to increase of the Kalman gain ***K***_***s***_, which means that the eigenvalues can change more quickly as the fiber tracking encounters changes in the data.

#### 2.2.5 seeds per voxel

The number of seeds per voxel is the number of times tractography is initiated within each voxel in a seeding region. If it satisfies the starting thresholds, each seed will trace out a fiber. Therefore, using more seeds usually generates more fibers. The choice of a value for this parameter depends on the voxel size of the data (if voxels are larger, more seeds will be needed within each) and on the machine used to run the UKF tractography, because a larger number of seeds will consume more computational time. In our experiments, the two patient datasets have voxel sizes of 2 × 2 × 3 *mm*^3^ and 2 × 2 × 2.6 *mm*^3^ (in-plane voxel sizes interpolated to 1 × 1 *mm*^2^ on the scanner), and one seed per voxel produced a number of traced fibers that was adequate to demonstrate the performance of UKF tractography.

### 2.3 Phantom data generation

In order to have a preliminary understanding of UKF tractography performance in edematous brain tissue in a relatively simplified situation, we started our experiments with synthetic (digital) phantom consisting of parallel fibers with added edema. The phantom was generated as synthetic DW-MRI data, using fractional anisotropy (FA) values and mean diffusivity (MD) values to characterize normal white matter and edema. Outside the simulated edema, the FA and MD values were in the range of normal white matter (Pierpaoli et al., 1996), while inside the simulated edema, the FA and MD values were in the range of peritumoral edematous brain (Sinha et al., 2002, Holodny and Ollenschlager, 2002, Lu et al., 2003).

The phantom data model combined a full diffusion tensor (to model normal white matter) and a spherical compartment (to model edema). Parallel tracts ran the length of each phantom, whose size was 150 *mm* × 110 *mm* × 110 *mm* with voxel dimensions of 2 *mm* × 2 *mm* × 2 *mm*. In the center of each phantom, a region of edema was simulated. Synthetic DW-MRI data was generated using 81 gradient directions uniformly spread on the hemisphere. The form of the signal model was:

(8) $$S_{i}/S_{0}=(1-\omega )e^{-bu_{i}^{T}D_{wm}u_{i}}+\omega e^{-bu_{i}^{T}d_{fw}Iu_{i}}$$

where *S*_*i*_ is the signal measured after the application of diffusion-sensitizing gradient in the unit direction of *u*_*i*_, *S*_0_ is the signal in the absence of diffusion sensitization, *ω* is the proportion of unweighted signal from the isotropic compartment, *D*_**wm**_ is the diffusion tensor to delineate the local microstructure of white matter and *d*_fw_ is the apparent diffusion coefficient (ADC) of the isotropic compartment. The free-water model is used to approximate the additional liquid (containing protein, water, etc.) in edema. In our model, *b* = 1000 *s*/*mm*^2^, *D*_**wm**_ = *λ*_1_*n*_*x*_*n*_*x*_^*T*^ + *λ*_2_*n*_*y*_*n*_*y*_^*T*^ + *λ*_3_*n*_*z*_*n*_*z*_^*T*^ where *λ*_1_ = 1100 × 10^− 6^ *mm*^2^/*s*,*λ*_2_ = *λ*_3_ = 450 × 10^− 6^ *mm*^2^/*s*, and *d*_fw_ = 3000 × 10^− 6^ *mm*^2^/*s*. The isotropic signal weight *ω* varied along the long axis of the phantom, from 0 in the two ends to 0.65 in the middle, simulating edematous brain tissue in the phantom's center. The diffusion MRI signal is influenced by Rician distributed noise (Gudbjartsson and Patz, 1995, Aja-Fernández et al., 2008, Rodrigues et al., 2010). To make the phantom more realistic, noise was applied to each diffusion signal *S*_*i*_ such that the noisy signal *S*_*i*_*′* complied with Rician distribution *f*(*S*_*i*_*′*|*S*_*i*_,*σ* = *S*_*i*_/15), which suggests that the signal-to-noise ratio (SNR) was around 15.

**Fig. 1:**
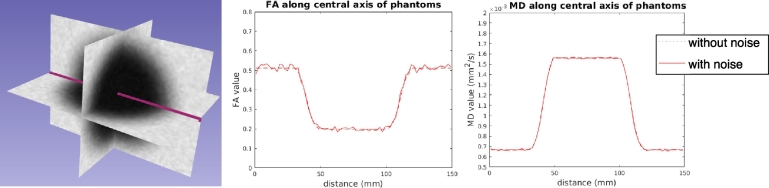
Synthetic data phantom. Left: grayscale FA images in 3D show the location of the simulated edema (the dark region in the phantom center). The simulated fiber tracts run anterior-posterior, in the orientation of the pink line. Center: FA values are plotted along the central axis of the phantom (this axis is shown as a pink line in the leftmost image). Right: The MD values are plotted along the central axis of the phantom.

One phantom was generated, where the synthetic edematous fibers had minimum FA values of 0.2 and maximum MD values of 1560 × 10^− 6^ *mm*^2^/*s*, respectively (Fig. 1). Tractography processing was performed in the phantom dataset using 3D Slicer (http://www.slicer.org, version 4) (Fedorov et al., 2012) via the SlicerDMRI project (http://dmri.slicer.org) and using UKF tractography as described below.

### 2.4 Patient data acquisition and processing

**Fig. 2:**
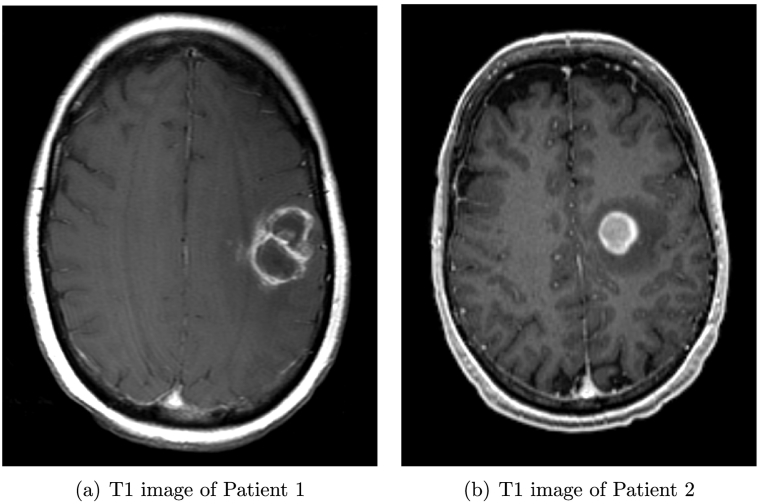
(a) Axial image from Patient 1 with a left fronto-parietal glioblastoma multiforme tumor and peritumoral edematous zone. (b) Axial image from Patient 2, with two metastatic lesions of unclassified pleomorphic sarcoma in the left frontal lobe and peritumoral edema. Gadolinium was given before the MRI scans.

To illustrate and test clinically relevant parameters of UKF tractography, we retrospectively selected two brain tumor patients with peritumoral edema affecting different structures: the arcuate fasciculus (AF) in one patient (Patient 1) and the corticospinal tract (CST) in the other (Patient 2). The two patients were selected for inclusion in this study from a data repository of neurosurgical patients who have undergone diffusion imaging at Brigham and Women's Hospital. The study was approved by the Partners Healthcare Institutional Review Board, and informed consent had been obtained from all participants prior to scanning. Patient 1 had a glioblastoma multiforme tumor with a peritumoral edematous zone in the vicinity of the language cortex, affecting the AF. Patient 2 had two metastatic lesions of unclassified pleomorphic sarcoma in the left frontal lobe, affecting the CST, and presented with right upper extremity weakness. Their axial T1-weighted image slices are illustrated in Fig. 2. MR images were obtained using a 3-Tesla scanner (EXCITE Signa scanner, GE Medical System, Milwaukee, WI, USA) with Excite 14.0, using an 8-channel head coil and array spatial sensitivity encoding technique (ASSET). Diffusion weighted images were acquired using EPI with 8 channel head coil and ASSET (TR = 14000 *ms*, TE = 75.4 *ms*, 31 gradient directions with a b-value of 1000 *s*/*mm*^2^, 1 baseline (b=0) image, FOV = 25.6 *cm*, matrix = 128 × 128, 44 and 52 slices, voxel size = 2 × 2 × 3 *mm*^3^ and 2 × 2 × 2.6 *mm*^3^ for the two datasets). The in-plane voxel sizes were interpolated to 1 × 1 *mm*^2^ on the scanner. fMRI images (used for quantifying the sensitivity of the tractography) were acquired using T2-weighted EPI with a birdcage coil (TR = 2000 *ms*, TE = 75.4 *ms*, FOV = 24 *cm*, matrix = 80 × 80, 27 axial slices, voxel size = 2 × 2 × 4 *mm*^3^). For Patient 1, task-based fMRI was obtained using an antonym task, and for Patient 2, three motor tasks were employed: lip pursing, foot tapping, and hand clenching.

3D Slicer was used to convert the raw data from DICOM format into NRRD format using DWIConvert (Matsui, 2014). DTIPrep (http://www.nitrc.org/projects/dtiprep) was used to perform quality control (Liu et al., 2010), which included artifact correction/removal as well as eddy-current and head motion artifacts correction by registration to the baseline image. A binary brain mask for each patient dataset was computed in 3D Slicer. Tractography processing was performed in several experiments as described below.

## 3 Results

We performed several experiments in phantom and patient data, both to evaluate the performance of UKF tractography in edema, and to investigate its strengths and limitations.

### 3.1 Phantom experiments

#### 3.1.1 UKF tractography versus streamline tractography in the phantom

**Fig. 3:**
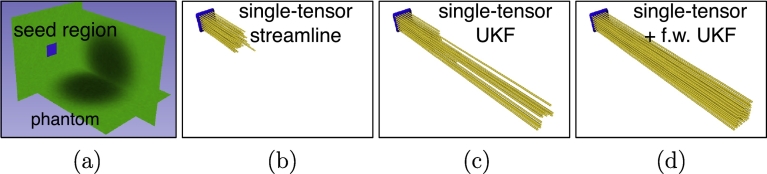
Recovery of simulated edematous fiber tracts. Three tractography methods were seeded in the synthetic edema phantom with minimum FA of 0.2. (a) A phantom containing parallel fibers running anterior-posterior (indicated by green color) with a region of synthetic edema in the phantom center. Simulated edematous tracts were recovered (yellow fibers) using default parameters for tractography. (b) The single-tensor streamline tractography that used independent single-tensor estimation at each voxel (least-squares) followed by Runge-Kutta order two integration for fiber tracking in 3D Slicer. (c) and (d) The two UKF methods (single tensor with and without free water model) that performed model estimation during tracking, using a Kalman filter.

First, we compared the performance of standard single-tensor streamline tractography (in 3D Slicer), single-tensor UKF tractography with and without the inclusion of free water model on our phantom (Fig. 3), using default parameters. The FA stopping criterion for the three methods was set the same as the lowest FA in our phantom: 0.2. The GA stopping criterion, which is specific to UKF tractography, was left at the default value of 0.1. The three tractography methods were all seeded at the same location, one end of the phantom.

In this simple experiment, the single-tensor UKF tractography with the inclusion of free water model performed more successfully than the other methods. The single-tensor streamline tractography cannot trace through the synthetic edema, and the single-tensor UKF tractography can only trace through the edema partly. Under the same stopping criteria, the single-tensor UKF tractography with free water model can fully trace through the edema reaching the other end of the phantom, which implies that the orientation of the fibers can be better retained in the tensor estimation when the additional free water model is included. It is clear that the edematous fibers need to be modeled by a more sophisticated model than the single-tensor model. The following experiments investigate the two-tensor UKF tractography and the effects of seeding regions and parameters on the output.

#### 3.1.2 Different seeding regions in the phantom

**Fig. 4:**
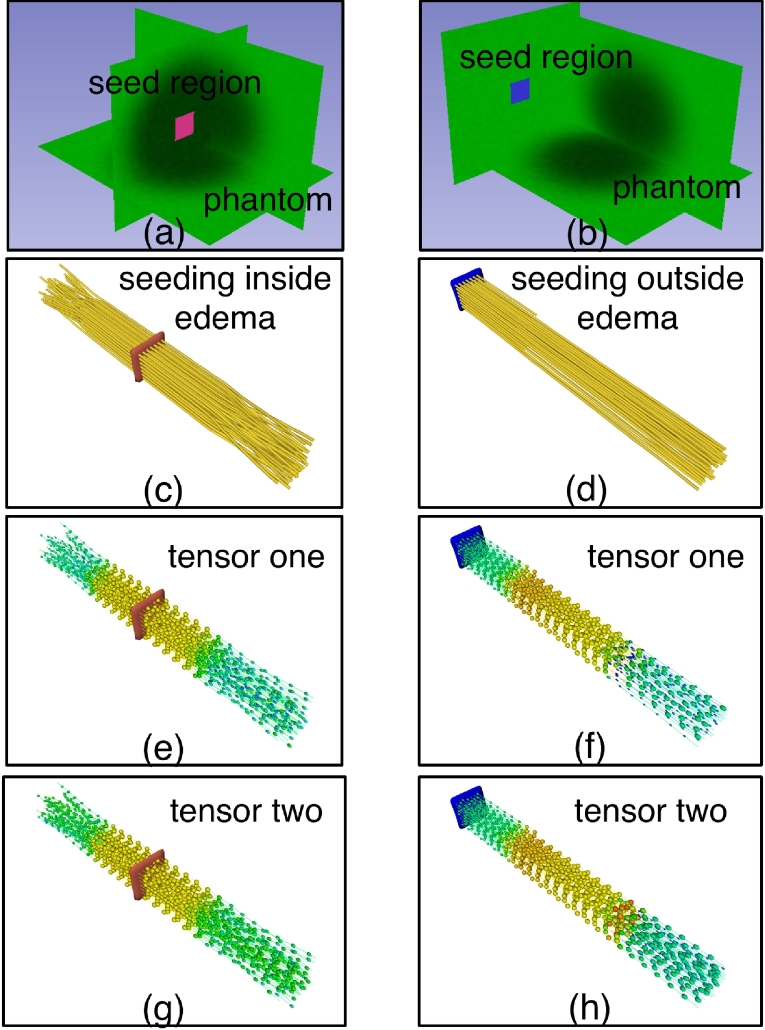
Tractography seeding scenarios for tracking through edema. Left column: two-tensor UKF tractography was seeded inside synthetic edema (pink seed region). Right column: two-tensor UKF tractography was seeded within simulated healthy white matter (blue seed region). (c) and (d): Traced fibers shown in yellow. (e) and (f): Tensor one that is the tensor followed during fiber tracking. The calculated tensor model is displayed along the fibers as ellipsoids colored by FA. Higher FA is green and blue, while lower FA (such as that in the edema) is orange.

Next we evaluated the performance of two-tensor UKF tractography when seeded from different regions. Two representative regions were chosen as seeding regions in the phantom, one at the end of the phantom and outside of the central edema as above, and the other at the center of the phantom and inside of the edema (Fig. 4). These were chosen to mimic two possible clinical seeding scenarios for tracking through edema in patients with brain tumors. Two-tensor UKF tractography with the FA stopping criterion of 0.16 was applied to each of these seed regions, and the other parameters were set as the default (Table 1). We lowered the FA stopping criterion here to get more fibers traced through the synthetic edema.

The tractography results show that the tract orientation was better established when tracking started outside of the synthetic edema, where the anisotropy was high. We also found that when seeded outside of the edema, in the fibers that were traced through the edema, the two tensors started diverging: one tensor retained its orientation and the other tensor became more round.

### 3.2 Patient data experiments

Next, we assessed the performance of UKF tractography in retrospective neurosurgical patient data. We performed experiments to compare one-tensor and two-tensor UKF tractography results, to investigate the effect of different seeding regions and to assess the effect of tractography parameters in clinical patient datasets.

#### 3.2.1 Comparison of one-tensor and two-tensor UKF

**Fig. 5:**
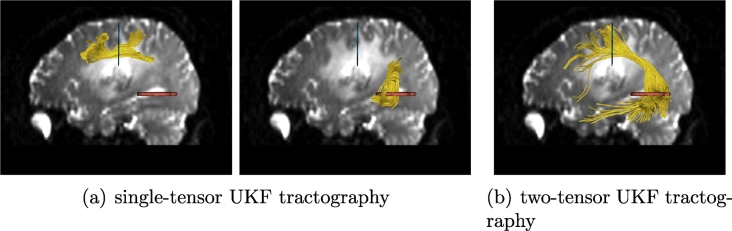
Comparison of single-tensor (a) and two-tensor (b) UKF tractography in the arcuate fasciculus of Patient 1. Two ROIs were applied to select the AF anatomy from the whole brain tractography. However, no fibers were found connecting the two ROIs using the single-tensor UKF tractography. To assess if the AF could be partially traced, we employed each ROI separately (left and center images), in conjunction with expert removal of fibers that appeared not to form part of the AF such as short U fibers.

**Fig. 6:**
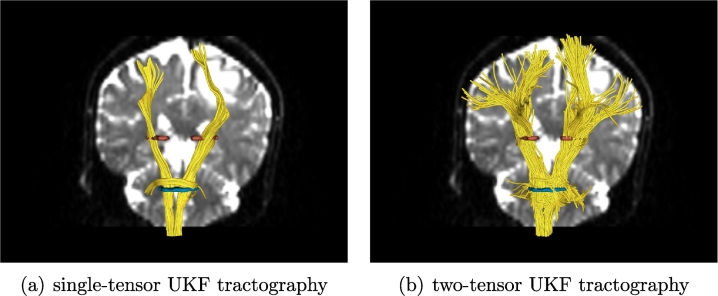
Comparison of single-tensor (a) and two-tensor (b) UKF tractography in the corticospinal tract of Patient 2. Two ROIs were applied to select the CST anatomy from the whole brain tractography. The two-tensor method (right image) can track more fibers for depiction of lateral connections.

In the phantom results, fibers can be traced through edema when using single-tensor UKF, whereas more typical single-tensor streamline tractography cannot. We have recently shown that two-tensor UKF tractography can trace a higher volume of the AF and the CST affected by edema, when compared to single-tensor streamline (Chen et al., 2015, Chen et al., 2016). However, this leaves open the question of if the differences in tractography are due to the UKF method or to the two-tensor model. For this reason, we began the experiments in patient data by comparing the performance of single-tensor UKF to two-tensor UKF tractography in patient datasets 1 and 2 (Fig. 5, Fig. 6). In these experiments, tractography was seeded throughout the entire brain using all default parameters of the algorithm (note that parameters will be tested below). Regions of interest (ROIs) were drawn to select the AF in Patient 1 and the CST in Patient 2. The results in AF and CST, where crossing fibers are known to affect the ability to trace the full structure, clearly demonstrate that the combination of the two-tensor model and the UKF tractography provides more fibers that can be traced through the edema, as shown in Fig. 5, Fig. 6. For this reason, in the rest of the experiments we focused solely on the two-tensor UKF method.

#### 3.2.2 Different seeding regions in patient data

Unlike our synthetic phantoms that had only one fiber tract orientation everywhere, the fiber tract anatomy of the brain varies with location. UKF tractography offers a causal estimation of the local structure along the fiber, which suggests that the initial seeding points may have a great influence on the output fibers. Therefore, different seeding regions for UKF tractography were tested to demonstrate the different influences. Several seeding regions were tested in each patient's data, using the two-tensor UKF tractography with default parameters. Seeding regions were defined along the AF and CST fiber tracts: we used patient-specific results from whole-brain tractography and fiber tract selection (above) as guidance while defining these regions, to ensure that the structure was expected to pass through them. Like in the phantom experiments, we chose seed regions that were both relatively affected and unaffected by edema for comparison, and we recorded the single-tensor FA value range within the seed regions.

**Fig. 7:**
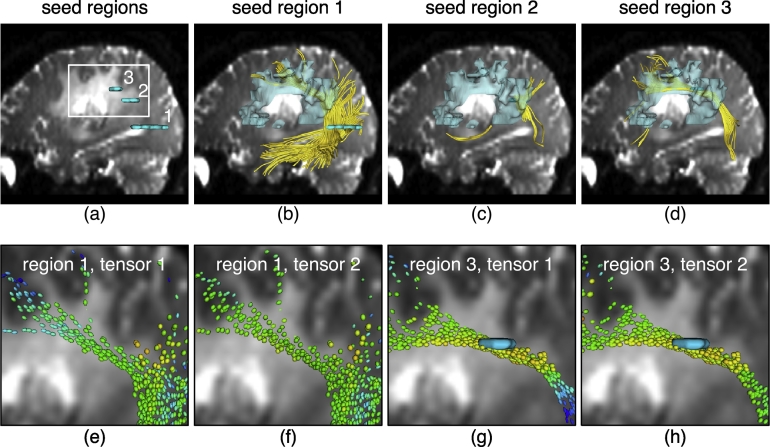
Two-tensor UKF tractography traverses edema but is affected by initial seeding location in Patient 1. In all views, the background anatomical images are the diffusion baseline images from Patient 1. Tensors are colored by FA, where blue represents a higher FA and orange/yellow is a lower FA. (a)-(d): Tractography (yellow fibers) is seeded from three different regions (cyan) within the arcuate fasciculus (AF). Seed region 1 is outside of the edema, while regions 2 and 3 are inside the edema. The translucent model represents the edematous region delineated by a clinical expert. (e) and (f): Zoomed-in views that show the two-tensor model when seeded in region 1. (g) and (h): Zoomed-in views that show the two-tensor model when seeded in region 3.

For Patient 1, seed region 1 was outside of the edematous brain tissue (FA 0.3–0.5), while regions 2 and 3 (FA 0.2–0.35) were inside the edema (Fig. 7). This edema had higher FA than our simulated edema phantom, so successful tracking could be expected even with default parameters. But though all three seed regions were located within the expected trajectory of the AF, the seeding results differed highly across seed regions, and results from only two regions traced a C-shaped AF structure. Crucially, the apparent distance of the AF from the tumor differed across seed regions. Seeding from region 3, the method showed the AF closest to the tumor margin. Note that the tensor 1 is oriented parallel to the tract (its major eigenvector was followed) while tensor 2 has a more arbitrary orientation, and it may model fiber crossings and/or be more in influenced by edema. It is apparent that the two-tensor model produced similar FA in the two tensors in some locations along the tracts, and in other locations the tensor 1 FA is higher (more blue). The variability in seeding results indicates that more robust seeding is necessary to assess the relationship of AF and tumor. The distance to the tumor border is important for surgical planning, so we recommend that a more stable seeding method (such as seeding in the whole brain or a large region) should be employed to find the AF for clinical research or clinical use.

**Fig. 8:**
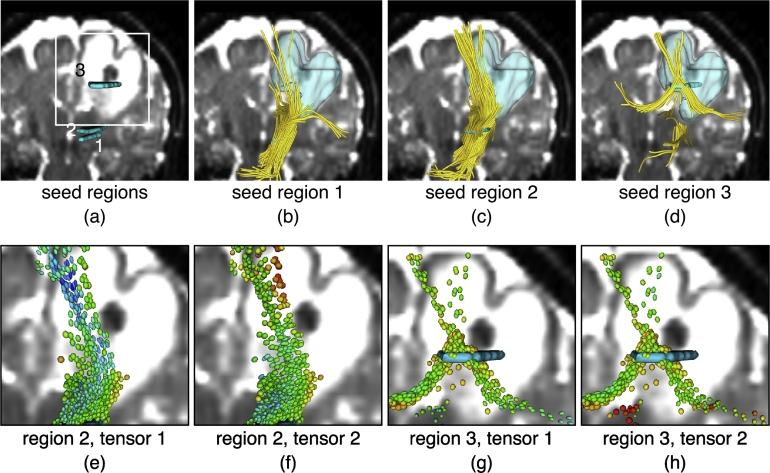
Two-tensor UKF tractography traverses edema but is affected by initial seeding location in Patient 2. In all views, the background anatomical images are the diffusion baseline images, shown at a location behind the fibers. Tensors are colored by FA, where blue represents a higher FA and orange/yellow is a lower FA. (a)–(d): Tractography (yellow fibers) is seeded from three different regions (cyan) within the corticospinal tract (CST). Regions 1 and 2 are outside of the edema, while region 3 is inside the edema. The translucent model represents the edematous region delineated by a clinical expert. (e) and (f): Zoomed-in views that show the two-tensor model when seeded in region 2. (g) and (h): Zoomed-in views that show the two-tensor model when seeded in region 3.

For Patient 2, seed regions 1 (FA 0.4–0.6) and 2 (FA 0.3–0.6) were near the boundary but outside of the edema, while seed region 3 (FA 0.05–0.2) was inside the edema (Fig. 8). All seed regions were within the expected trajectory of the CST, but again the results were sensitive to the choice of seedpoint. Seeding in the region with the most severe edema was less successful at depicting the CST, though some tracts crossing through this region were able to be traced. Note that the tensor 1 is oriented parallel to the tract (its major eigenvector was followed) while tensor 2 has a more arbitrary orientation, and may model fiber crossings and/or be more influenced by edema. Similar to the result in AF, in several locations the FA of tensor 1 was higher (more blue) than the FA of tensor 2 along the tracts. This indicates that in these regions, the second tensor was representing the edema to a greater extent than the first tensor. When seeded in region 3, the two tensors in the edema were almost round (relatively low orange/yellow FA), which made the tractography uncertain initially.

In all seeding experiments, tracts could be traced through edema. Overall, the results in these two patients indicate that the second tensor was somewhat more likely to represent edema (have lower FA) than the first tensor, which is the one followed during fiber tracking. Furthermore, the results indicate that small or single-slice seed regions can lead to variable depiction of anatomy with two-tensor UKF tractography. Thus for robust depiction of anatomy, clinical users of UKF should employ large or multiple seed regions. The following experiments, therefore, focus on whole-brain tractography seeding, followed by anatomical selection of tracts of interest.

#### 3.2.3 Whole brain seeding with different parameter settings in patient data

We performed final experiments to verify that whole brain seeding can more completely depict brain white matter anatomy and to investigate how the parameters influence output tractography in patient datasets. Parameters tested included default values as well as values expected to increase the number and length of fibers: FA stopping criteria of 0.1 and 0.15, GA stopping criteria of 0.05, 0.075 and 0.1, *q_L_* values of 50, 100 and 200 (where higher values than default are used to test performance in edema), and the use of a free water model were tested. Also, their combinations were tested. For each parameter setting experiment, two-tensor UKF tractography was seeded within the whole brain of each patient dataset, using the binary brain mask as a seed region. As above, the ROIs (Fig. 5, Fig. 6) were used to select the AF in Patient 1 and the CST in Patient 2.

**Fig. 9:**
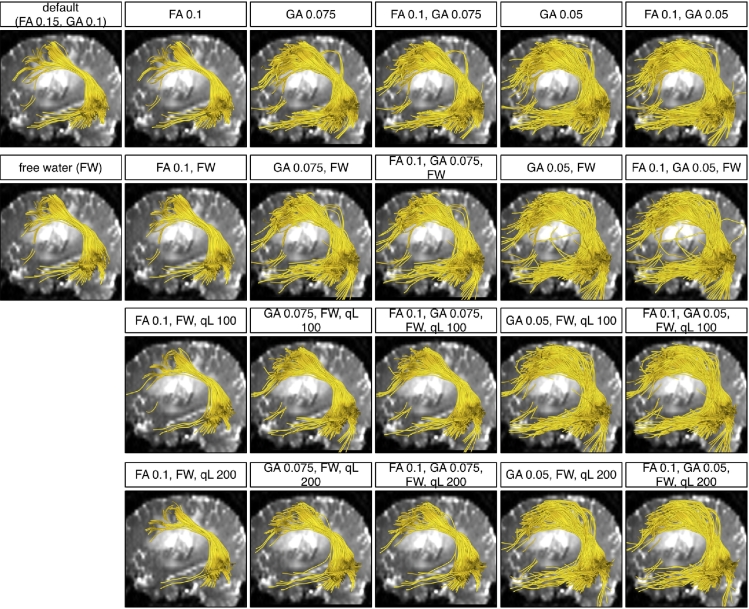
Two-tensor UKF tractography reconstructions of the AF from 22 different parameter settings in Patient 1. The background image is a diffusion *b*_0_ image, medial to the AF. Six FA and GA threshold settings are demonstrated in columns. Four *q*_*L*_ settings are shown in rows. The top row displays the results without including the free-water model and the three bottom rows include it.

For Patient 1, traced fibers from 22 different parameter settings are shown in Fig. 9. First note that reconstruction of the AF from whole brain seeding was apparently more complete and corresponded better to the usual anatomical descriptions of the AF than the previous results from the three seed regions (Fig. 7). Also note that the most crucial parameter to increase tracking through edema is the GA threshold. The FA threshold was not so crucial as in the phantom experiments, because the edematous brain tissue in Patient 1 was observed to have FA values above the FA stopping criteria of 0.15. Additionally, the FA stopping threshold applies only to tensor one, the tensor being tracked, which we saw may have higher FA than tensor two. Inclusion of the free-water model did not have a large effect on the reconstruction in these two patient datasets. A higher *q*_*L*_ removed the outlier fibers whose orientations had changed rapidly.

**Fig. 10:**
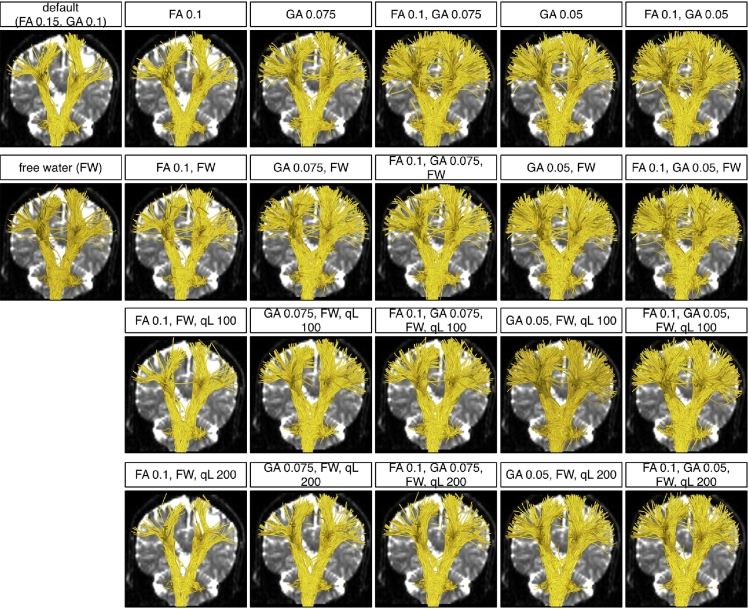
Two-tensor UKF tractography reconstructions of the CST from 22 different parameter settings in Patient 2. The background image is a diffusion *b*_0_ image, posterior to the CST to avoid occluding fibers. Six FA and GA threshold settings are demonstrated in columns. Four *q*_*L*_ settings are shown in rows. The top row displays the results without including the free-water model and the three bottom rows include it.

For Patient 2, traced fibers from 22 different parameter settings are shown in Fig. 10. Similar to the results from Patient 1, the tractography seeded from the whole brain reconstructed a larger volume of CST than the tractography seeded from local seed regions. In this reconstruction, FA and GA thresholds were both crucial for tracing the edematous fibers, as the FA values in the edematous region were observed to be lower than those in Patient 1. Therefore, a lower FA stopping criteria was necessary. A higher *q*_*L*_ also removed many outlier fibers which had unreasonably crossed between hemispheres.

#### 3.2.4 Evaluation of parameter settings with respect to fMRI

We quantitatively assessed the tractography results from different parameter settings using a so-called coverage measure (Baumgartner et al., 2012), which allowed us to quantify the sensitivity of the tractography for connecting to a patient-specific fMRI activation. For a fiber tract bundle fiber_*i*_, this measure *C*_fiber_*i*__ is defined by the ratio of the voxels *V*_fiber_*i*__ passed by fiber_*i*_ in its corresponding target region *V*_target_*i*__ to the total number of voxels in *V*_target_*i*__, which is $C_{{\text{fiber}}_{i}}=\frac{V_{{\text{fiber}}_{i}}}{V_{{\text{target}}_{i}}}$. AF is known to connect the Broca's area (for speech production) with the Wernicke's area (for speech comprehension) and CST is known to connect the area of motor cortex with the spinal cord. Additionally, the corticobulbar tract connects the face motor area to the brainstem, and we consider it as part of CST for the purposes of this experiment. fMRI datasets from Patients 1 and 2 (from language and motor tasks, respectively) were processed clinically and thresholded by an expert, then registered to the baseline volumes of their corresponding diffusion MRI datasets. The resulting activation regions were used to locate the putative Broca's area of Patient 1 and the face/hand/foot areas of motor cortex of Patient 2, which were respectively the target regions of AF and CST.

As is usual in surgical planning, the fMRI regions were not anatomically constrained to the gray matter, but were rather determined using expert thresholding, which is considered to be the gold standard method for single-subject presurgical fMRI (Norton et al., 2014). We note that in tractography for surgical planning, fMRI activation regions are often enlarged for tract selection (Golby et al., 2011, Schonberg et al., 2006, Kleiser et al., 2010) because especially in the presence of edema, white matter tractography may not fully reach the cortex.

**Fig. 11:**
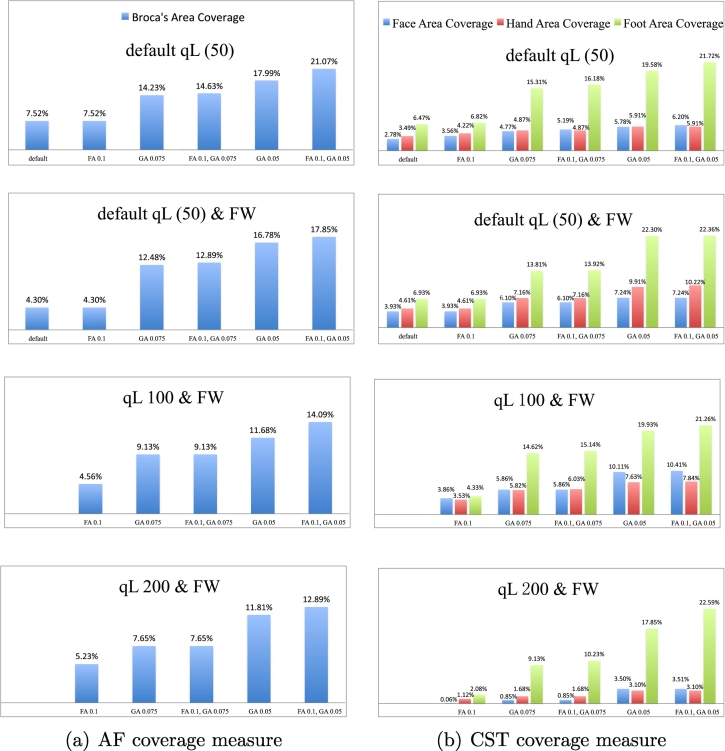
Quantitative measures of tractography sensitivity based on patient-specific fMRI demonstrate the effect of varying UKF tractography parameters. The coverage ratio is measured as the percentage of the target fMRI activation that is reached by the fiber tracts. (a) Reconstructed AF coverage ratio of Broca's area from all parameter settings experiments in the data of Patient 1. (b) Reconstructed CST coverage ratio of the face/foot/hand motor areas from all parameter settings experiments in the data of Patient 2.

The coverage measure results (Fig. 11) indicated that two-tensor UKF tractography was more sensitive to the GA stopping threshold than to the FA threshold. But when the GA threshold was set lower than 0.075, the results were no longer so sensitive to the GA threshold. In addition, the inclusion of the free water model did not have a clear effect on the tractography sensitivity as quantified by the coverage measure in these two patient datasets, though in some cases it increased the sensitivity. Increasing the *q*_*L*_ value did slightly decrease the sensitivity of the tractography results. Also, the UKF tractography was apparently most sensitive in the foot region, followed by the hand then the face regions.

It is important to note that the fMRI activation region cannot be perfectly regarded as the ground truth of tractography. Activation region thresholding of fMRI and registration between fMRI and diffusion MRI can all affect the coverage measure. It is also expected that the co-location of AF and language activations will not be perfect (Diehl et al., 2010) as the activation areas are known to be anatomically variable. Therefore, the coverage measure is used here for assessing the tractography sensitivity under different parameter settings for this small study of two patients, and should not be interpreted as a validation of the tractography results.

## 4 Discussion and conclusions

Our overall findings from applying UKF tractography in retrospective neurosurgical patient data were as follows. When seeded from the whole brain, the two-tensor UKF tractography could reconstruct a more complete WM anatomy in the edema, in comparison with seeding from small regions. We note that it has been demonstrated in healthy subjects that the “brute-force” whole-brain seeding approach, in combination with multiple selection ROIs, improves DTI-based tractography when compared with local single-ROI seeding (Huang et al., 2004). Seeding approaches have also been studied in patients with brain tumors, where whole-brain seeding or large area seeding was found to be more stable (Radmanesh et al., 2015, Niu et al., 2016, Golby et al., 2011).

For the parameter settings, FA and GA thresholds determined where the fiber tracking stopped, so they were usually crucial parameters for the reconstruction of edematous fibers. However, no fixed FA and GA thresholds could be perfectly applied to every patient (see following discussion). Inclusion of the free-water model did not have a clear effect on the tractography in these two patient datasets, while increasing the *q*_*L*_ value could avoid some rapid changes of the orientations of the fibers, reducing outlier (false positive) fibers.

Most studies of tractography in surgical planning so far have focused on increasing sensitivity to trace the anatomically expected full extent of tracts of interest (Chen et al., 2015, Chen et al., 2016, Mandelli et al., 2014). We believe it would be ideal to consider false positives in a future investigation; however, defining the regions in the cortex that should not receive language or motor connections is currently somewhat challenging. This has recently inspired the design of synthetic brain phantoms where ground truth is known, such as the phantom used in the ISMRM Tractography Challenge^1^ (Neher et al., 2014). Defining false positive connections in the human brain is challenging, especially in the case of language connections, because recent work has demonstrated the possibility of widespread language activity over the entire cortex (Huth et al., 2016). False positive motor connections are also not simply defined, as corticospinal motor connections were recently demonstrated to multiple regions including primary motor, primary somatosensory, and dorsal premotor cortices, plus the supplementary motor area (Seo and Jang, 2013). However, we did employ one well-established method for rejecting known false positives that cross the midline, which are much easier to identify (Behrens et al., 2007).

We partially addressed the lack of ground truth by building a synthetic digital phantom with simulated edema. Though our phantom was intended to provide insight for two-tensor UKF tractography, and was not realistic in terms of crossing fibers or any other feature of neuroanatomy, the results in the phantom were qualitatively similar to those in patient data. Therefore, our phantom can be considered to be a simplified test dataset for tractography through edematous brain. It is of future interest to more realistically model anatomical fiber tracts affected by edema. At the present time, however, the problem of how to design realistic phantoms for diffusion MRI and for tractography is currently under active study (Côté et al., 2013, Neher et al., 2014, Neher et al., 2015, Esteban et al., 2016), but is not yet solved, even for neuroanatomical modeling of healthy subjects' data.

Fitting the two-tensor model to the diffusion signals is an ill-posed problem; UKF tractography is a causal tractography method, so each step's model fitting is partly determined by the signal configuration of previous steps. Likely for this reason, we have observed that UKF tractography is relatively sensitive to to the region chosen for seeding (Fig. 7, Fig. 8). In contrast, single-tensor streamline tractography generally will produce more or less the same structure when seeded at different locations along the tract, since the model fitting at each location is independent. Regarding the performance of the two-tensor model when tracking through edema, we have seen that the fitted two tensors may diverge into a spherical one and an elliptical one when tracking through edematous brain or simulated edema (Fig. 4, Fig. 7, Fig. 8). But this does not always happen; what we can say is that the two tensors together will model the data and that this provides increased flexibility for tracking through edema relative to a single-tensor model with fewer parameters.

Based on our experimental results, we provide suggested procedures for employing UKF tractography to reconstruct edematous WM. First, UKF tractography should be seeded from the whole brain. We have previously empirically determined across multiple datasets (O’Donnell et al., 2017) that if the voxels are 2 × 2 × 2 *mm*^3^ or larger, multiple seed points should be used per voxel. Our datasets (which had been interpolated to higher resolution on the scanner) had voxel sizes of 1 × 1 × 2.6 *mm*^3^ and 1 × 1 × 3 *mm*^3^. Secondly, for parameter settings, our results show that reducing the GA and FA stopping thresholds (below the default settings of GA of 0.1 and FA of 0.15) will increase the tractography sensitivity, producing apparently larger fiber tracts. In general, UKF tractography is more affected by the GA threshold than the FA threshold. The default *q*_*L*_ of 50 can provide good results. After whole brain seeding of tractography, clinically relevant fiber tract(s) of interest may be identified by an expert or an automated method. We note that this initial evaluation focused on two patients. In the future, UKF, and especially the free water model, will benefit from more testing in multiple patients with different tumor types, levels of edema, and relationships between tract and tumor.

These results are based on our dataset, and they provide intuition into the parameters for any reader interested in trying this method in his or her own dataset. The proposed parameters should be broadly useful for data acquired around *b* = 1000 *s*/*mm*^2^ with 30 or more gradient directions, similar to our dataset. We note that the UKF tractography method expects a certain level of noise in the data depending on the settings of the q parameters, so “good” settings for these parameters for different data (such as that with a higher b-value) can be determined by experiments similar to those we have performed here.

Although this work demonstrates that two-tensor UKF tractography with appropriate seeding/parameter settings can somewhat reconstruct more edematous WM structures, we have not totally addressed the challenge of tracking through edema. Increasing sensitivity may lead to increased false positive tracts. The particular two-tensor model, while it fits the data well, was not biophysically designed in any way to represent edema. The biophysical free water model can represent CSF, but the diffusivity of edema is not necessarily expected to equal that of CSF. Some groups also attempted to address this challenge in the imaging/scanner level with other fiber orientation estimation/modeling methods. Zhang et al. (2013) compared generalized q-sampling based tractography with DTI-based WM mapping in edematous regions. They demonstrated that DTI-based tractography missed several fiber tracts in 5 cases in comparison with those from generalized q-sampling imaging (GQI) data. Another study by Kuhnt et al. (2013) demonstrated that high angular resolution diffusion imaging (HARDI)-compressed sensing (CS) technique was able to visualize some peritumoral WM fiber bundles that DTI could not achieve. McDonald et al. (2013) have shown that by applying restricted spectrum imaging (RSI), using multiple diffusion weightings to remove volume fraction associated with edema, the quantification and visualization of white matter tracts in peritumoral regions could be improved. A recent study by Abhinav et al. (2015) illustrated a more accurate depiction of peritumoral tracts in their preliminary experiments, employing a so-called high-definition fiber tractography (HDFT). The pathology of brain tumors is patient-specific. Therefore, UKF, and especially the free water model, will benefit from additional future investigation in multiple patients with different tumor types, levels of edema, and relationships between tract and tumor.

In conclusion, the main contribution of this study is providing an insight into how UKF tractography works, with a two-tensor model, to somewhat address the challenge of edematous WM reconstruction. We built a synthetic digital edema phantom to simplify the anatomy of edematous brain tissues, such that we could have a preliminary understanding of the performance of UKF tractography in the setting of edema. We showed in retrospective neurosurgical patient data how different choices of seeding regions and parameter settings affected UKF tractography results. Furthermore, we have provided some guidance of how to use UKF tractography in neurosurgical planning research to increase sensitivity for fiber tracking through edema.

## Footnotes

1 http://www.tractometer.org/ismrm_2015_challenge/data

## References

[bb0005] Abhinav K., Yeh F.-C., Mansouri A., Zadeh G., Fernandez-Miranda J.C. (2015). High-definition fiber tractography for the evaluation of perilesional white matter tracts in high-grade glioma surgery. Neuro-oncology.

[bb0010] Aja-Fernández S., Alberola-López C., Westin C.-F. (2008). Noise and signal estimation in magnitude MRI and Rician distributed images: a LMMSE approach. Image Processing, IEEE Transactions on.

[bb0015] Akai H., Mori H., Aoki S., Masutani Y., Kawahara N., Shibahara J., Ohtomo K. (2005). Diffusion tensor tractography of gliomatosis cerebri: fiber tracking through the tumor. J. Comput. Assist. Tomogr..

[bb0020] Alexander A.L., Hasan K.M., Lazar M., Tsuruda J.S., Parker D.L. (2001). Analysis of partial volume effects in diffusion-tensor MRI. Magn. Reson. Med..

[bb0025] Basser P., Pajevic S., Pierpaoli C., Duda J., Aldroubi A. (2000). In vivo fiber tractography using DT-MRI data. Magn. Reson. Med..

[bb0030] Basser P.J., Pajevic S., Pierpaoli C., Duda J., Aldroubi A. (2000). In vivo fiber tractography using DT-MRI data. Magn. Reson. Med..

[bb0035] Basser P.J. (1995). Inferring microstructural features and the physiological state of tissues from diffusion-weighted images. NMR Biomed..

[bb0040] Basser P.J., Mattiello J., LeBihan D. (1994). MR diffusion tensor spectroscopy and imaging. Biophys. J..

[bb0045] Baumgartner C., Michailovich O., Levitt J., Pasternak O., Bouix S., Westin C., Rathi Y. (2012). A unified tractography framework for comparing diffusion models on clinical scans. CDMRI Workshop-MICCAI, Nice.

[bb0050] Behrens T., Woolrich M., Jenkinson M., Johansen-Berg H., Nunes R., Clare S., Matthews P., Brady J., Smith S. (2003). Characterization and propagation of uncertainty in diffusion-weighted MR imaging. Magn. Reson. Med..

[bb0055] Behrens T.E., Berg H.J., Jbabdi S., Rushworth M., Woolrich M. (2007). Probabilistic diffusion tractography with multiple fibre orientations: what can we gain?. Neuroimage.

[bb0060] Berman J.I., Berger M.S., Mukherjee P., Henry R.G. (2004). Diffusion-tensor imaging-guided tracking of fibers of the pyramidal tract combined with intraoperative cortical stimulation mapping in patients with gliomas. J. Neurosurg..

[bb0065] Chen Z., Tie Y., Olubiyi O., Rigolo L., Mehrtash A., Norton I., Pasternak O., Rathi Y., Golby A.J., O’Donnell L.J. (2015). Reconstruction of the arcuate fasciculus for surgical planning in the setting of peritumoral edema using two-tensor unscented Kalman filter tractography. NeuroImage: Clinical.

[bb0070] Chen Z., Tie Y., Olubiyi O., Zhang F., Mehrtash A., Rigolo L., Kahali P., Norton I., Pasternak O., Rathi Y., Golby A.J., O’Donnell L.J. (2016). Corticospinal tract modeling for neurosurgical planning by tracking through regions of peritumoral edema and crossing fibers using two-tensor unscented Kalman filter tractography. Int. J. Comput. Assist. Radiol. Surg..

[bb0075] Conturo T.E., Lori N.F., Cull T.S., Akbuda E., Snyder A.Z., Shimony J.S., McKinstry R.C., Burton H., Raichle A.E. (1999). Tracking neuronal fiber pathways in the living human brain. Neurobiology.

[bb0080] Côté M.-A., Girard G., Boré A., Garyfallidis E., Houde J.-C., Descoteaux M. (2013). Tractometer: towards validation of tractography pipelines. Med. Image Anal..

[bb0085] Descoteaux M., Deriche R., Knosche T., Anwander A. (2009). Deterministic and probabilistic tractography based on complex fibre orientation distributions. Medical Imaging, IEEE Transactions on.

[bb0090] Diehl B., Piao Z., Tkach J., Busch R.M., LaPresto E., Najm I., Bingaman B., Duncan J., Lüders H. (2010). Cortical stimulation for language mapping in focal epilepsy: correlations with tractography of the arcuate fasciculus. Epilepsia.

[bb0095] Duffau H. (2014). The dangers of magnetic resonance imaging diffusion tensor tractography in brain surgery. World Neurosurg..

[bb0100] Esteban O., Caruyer E., Daducci A., Bach-Cuadra M., Ledesma-Carbayo M.J., Santos A. (2016). Diffantom: whole-brain diffusion MRI phantoms derived from real datasets of the human connectome project. Front. Neuroinform..

[bb0105] Farquharson S., Tournier J.-D., Calamante F., Fabinyi G., Schneider-Kolsky M., Jackson G.D., Connelly A. (2013). White matter fiber tractography: why we need to move beyond DTI: Clinical article. J. Neurosurg..

[bb0110] Fedorov A., Beichel R., Kalpathy-Cramer J., Finet J., Fillion-Robin J.-C., Pujol S., Bauer C., Jennings D., Fennessy F., Sonka M., Buatti J., Aylward S., Miller J.V., Pieper S., Kikinis R. (2012). 3D Slicer as an image computing platform for the quantitative imaging network. Magn. Reson. Imaging.

[bb0115] Feigl G.C., Hiergeist W., Fellner C., Schebesch K.-M.M., Doenitz C., Finkenzeller T., Brawanski A., Schlaier J. (2014). Magnetic resonance imaging diffusion tensor tractography: evaluation of anatomic accuracy of different fiber tracking software packages. World Neurosurg..

[bb0120] Fernandez-Miranda J.C. (2013). Editorial: beyond diffusion tensor imaging. J. Neurosurg..

[bb0125] Friman O., Farneback G., Westin C.-F. (2006). A Bayesian approach for stochastic white matter tractography. Medical Imaging, IEEE Transactions on.

[bb0130] Golby A.J., Kindlmann G., Norton I., Yarmarkovich A., Pieper S., Kikinis R. (2011). Interactive diffusion tensor tractography visualization for neurosurgical planning. Neurosurgery.

[bb0135] Gudbjartsson H., Patz S. (1995). The Rician distribution of noisy MRI data. Magn. Reson. Med..

[bb0140] Holodny A.I., Ollenschlager M. (2002). Diffusion imaging in brain tumors. Neuroimaging Clin. N. Am..

[bb0145] Huang H., Zhang J., van Zijl P., Mori S. (2004). Analysis of noise effects on DTI-based tractography using the brute-force and multi-ROI approach. Magn. Reson. Med..

[bb0150] Huth A.G., de Heer W.A., Griffiths T.L., Theunissen F.E., Gallant J.L. (2016). Natural speech reveals the semantic maps that tile human cerebral cortex. Nature.

[bb0155] Jellison B.J., Field A.S., Medow J., Lazar M., Salamat M.S., Alexander A.L. (2004). Diffusion tensor imaging of cerebral white matter: a pictorial review of physics, fiber tract anatomy, and tumor imaging patterns. Am. J. Neuroradiol..

[bb0160] Jones D.K., Simmons A., Williams S.C.R., Horsfield M.A. (1999). Non-invasive assesment of axonal fiber connectivity in the human brain via diffusion tensor MRI. Magn. Reson. Med..

[bb0165] Kaden E., Knösche T.R., Anwander A. (2007). Parametric spherical deconvolution: inferring anatomical connectivity using diffusion MR imaging. Neuroimage.

[bb0170] Kinoshita M., Yamada K., Hashimoto N., Kato A., Izumoto S., Baba T., Maruno M., Nishimura T., Yoshimine T. (2005). Fiber-tracking does not accurately estimate size of fiber bundle in pathological condition: initial neurosurgical experience using neuronavigation and subcortical white matter stimulation. Neuroimage.

[bb0175] Kleiser R., Staempfli P., Valavanis A., Boesiger P., Kollias S. (2010). Impact of fMRI-guided advanced DTI fiber tracking techniques on their clinical applications in patients with brain tumors. Neuroradiology.

[bb0180] Kuhnt D., Bauer M.H., Egger J., Richter M., Kapur T., Sommer J., Merhof D., Nimsky C. (2013). Fiber tractography based on diffusion tensor imaging compared with high-angular-resolution diffusion imaging with compressed sensing: initial experience. Neurosurgery.

[bb0185] Lazar M., Weinstein D.M., Tsuruda J.S., Hasan K.M., Arfanakis K., Meyerand M.E., Badie B., Rowley H.A., Haughton V., Field A., Alexander A.L. (2003). White matter tractography using diffusion tensor deflection. Hum. Brain Mapp..

[bb0190] Le Bihan D. (2003). Looking into the functional architecture of the brain with diffusion MRI. Nat. Rev. Neurosci..

[bb0195] Le Bihan D., Breton E., Lallemand D., Grenier P., Cabanis E., Laval-Jeantet M. (1986). MR imaging of intravoxel incoherent motions: application to diffusion and perfusion in neurologic disorders. Radiology.

[bb0200] Le Bihan D., Johansen-Berg H. (2012). Diffusion MRI at 25: exploring brain tissue structure and function. Neuroimage.

[bb0205] Le Bihan D., Poupon C., Amadon A., Lethimonnier F. (2006). Artifacts and pitfalls in diffusion MRI. J. Magn. Reson. Imaging.

[bb0210] Liu Z., Wang Y., Gerig G., Gouttard S., Tao R., Fletcher T., Styner M. (2010). Quality control of diffusion weighted images. SPIE Medical Imaging. International Society for Optics and Photonics.

[bb0215] Lu S., Ahn D., Johnson G., Cha S. (2003). Peritumoral diffusion tensor imaging of high-grade gliomas and metastatic brain tumors. Am. J. Neuroradiol..

[bb0220] Malcolm J., Shenton M., Rathi Y. (2010). Filtered multitensor tractography. Medical Imaging, IEEE Transactions on.

[bb0225] Mandelli M.L., Berger M.S., Bucci M., Berman J.I., Amirbekian B., Henry R.G. (2014). Quantifying accuracy and precision of diffusion MR tractography of the corticospinal tract in brain tumors: clinical article. J. Neurosurg..

[bb0230] Matsui J.T. (2014). Development of Image Processing Tools and Procedures for Analyzing Multi-Site Longitudinal Diffusion-Weighted Imaging Studies.

[bb0235] McDonald C.R., White N.S., Farid N., Lai G., Kuperman J.M., Bartsch H., Hagler D.J., Kesari S., Carter B.S., Chen C.C., Dale C. (2013). Recovery of white matter tracts in regions of peritumoral flair hyperintensity with use of restriction spectrum imaging. Am. J. Neuroradiol..

[bb0240] McGirt M.J., Chaichana K.L., Gathinji M., Attenello F.J., Than K., Olivi A., Weingart J.D., Brem H., Quiñones-Hinojosa A.R. (2009). Independent association of extent of resection with survival in patients with malignant brain astrocytoma: clinical article. J. Neurosurg..

[bb0245] Mori S., Crain B., Chacko V., van Zijl P. (1999). Three dimensional tracking of axonal projections in the brain by magnetic resonance imaging. Ann Neurol.

[bb0250] Neher P.F., Descoteaux M., Houde J.C., Stieltjes B., Maier-Hein K.H. (2015). Strengths and weaknesses of state of the art fiber tractography pipelines-a comprehensive in-vivo and phantom evaluation study using tractometer. Med. Image Anal..

[bb0255] Neher P.F., Laun F.B., Stieltjes B., Maier-Hein K.H. (2014). Fiberfox: facilitating the creation of realistic white matter software phantoms. Magn. Reson. Med..

[bb0260] Nimsky C. (2014). Fiber tracking-we should move beyond diffusion tensor imaging. World Neurosurg..

[bb0265] Nimsky C., Bauer M., Carl B. (2016). Merits and limits of tractography techniques for the uninitiated. Advances and Technical Standards in Neurosurgery.

[bb0270] Nimsky C., Ganslandt O., Hastreiter P., Wang R., Benner T., Sorensen A.G., Fahlbusch R. (2005). Preoperative and intraoperative diffusion tensor imaging-based fiber tracking in glioma surgery. Neurosurgery.

[bb0275] Niu C., Liu X., Yang Y., Zhang K., Min Z., Wang M., Li W., Guo L., Lin P., Zhang M. (2016). Assessing region of interest schemes for the corticospinal tract in patients with brain tumors. Medicine.

[bb0280] Norton I.H., Orringer D.A., Golby A.J. (2014). Image-guided neurosurgical planning. Intraoperative Imaging and Image-Guided Therapy.

[bb0285] O’Donnell L.J., Suter Y., Rigolo L., Kahali P., Zhang F., Norton I., Albi A., Olubiyi O., Meola A., Essayed W.I., Unadkat P., Ciris P.A., Wells I.I.I. W.M., Rathi Y., Westin C.-F., Golby A.J. (2017). Automated white matter fiber tract identification in patients with brain tumors. NeuroImage: Clinical.

[bb0290] Papadopoulos M., Saadoun S., Binder D., Manley G., Krishna S., Verkman A. (2004). Molecular mechanisms of brain tumor edema. Neuroscience.

[bb0295] Parker G.J., Alexander D.C. (2005). Probabilistic anatomical connectivity derived from the microscopic persistent angular structure of cerebral tissue. Philos. Trans. R. Soc., B.

[bb0300] Pasternak O., Assaf Y., Intrator N., Sochen N. (2008). Variational multiple-tensor fitting of fiber-ambiguous diffusion-weighted magnetic resonance imaging voxels. Magn. Reson. Imaging.

[bb0305] Pasternak O., Sochen N., Gur Y., Intrator N., Assaf Y. (2009). Free water elimination and mapping from diffusion MRI. Magn. Reson. Med..

[bb0310] Peled S., Friman O., Jolesz F., Westin C.-F. (2006). Geometrically constrained two-tensor model for crossing tracts in DWI. Magn. Reson. Imaging.

[bb0315] Pierpaoli C., Jezzard P., Basser P.J., Barnett A., Di Chiro G. (1996). Diffusion tensor MR imaging of the human brain. Radiology.

[bb0320] Pierpaoli C., Jones D. (2004). Removing CSF contamination in brain DT-MRIs by using a two-compartment tensor model. Proc. International Society for Magnetic Resonance in Medicine 12th Scientific Meeting ISMRM04.

[bb0325] Qazi A.A., Radmanesh A., O’Donnell L., Kindlmann G., Peled S., Whalen S., Westin C.-F., Golby A.J. (2009). Resolving crossings in the corticospinal tract by two-tensor streamline tractography: method and clinical assessment using fMRI. Neuroimage.

[bb0330] Radmanesh A., Zamani A.A., Whalen S., Tie Y., Suarez R.O., Golby A.J. (2015). Comparison of seeding methods for visualization of the corticospinal tracts using single tensor tractography. Clin. Neurol. Neurosurg..

[bb0335] Rathi Y., Gagoski B., Setsompop K., Michailovich O., Grant P.E., Westin C.-F. (2013). Diffusion propagator estimation from sparse measurements in a tractography framework. Medical Image Computing and Computer-Assisted Intervention-MICCAI 2013.

[bb0340] Rodrigues P., Prckovska V., Pullens W., Strijkers G., Vilanova A., ter Haar Romeny B. (2010). Validating validators: an analysis of DW-MRI hardware and software phantoms. Proc. Intl. Soc. Mag. Reson. Med.

[bb0345] Sanai N., Berger M.S. (2008). Glioma extent of resection and its impact on patient outcome. Neurosurgery.

[bb0350] Schonberg T., Pianka P., Hendler T., Pasternak O., Assaf Y. (2006). Characterization of displaced white matter by brain tumors using combined DTI and fMRI. Neuroimage.

[bb0355] Seo J., Jang S. (2013). Different characteristics of the corticospinal tract according to the cerebral origin: DTI study. Am. J. Neuroradiol..

[bb0360] Sinha S., Bastin M.E., Whittle I.R., Wardlaw J.M. (2002). Diffusion tensor MR imaging of high-grade cerebral gliomas. Am. J. Neuroradiol..

[bb0365] Talos I.-F., O’Donnell L., Westin C.-F., Warfield S.K., Wells I.I.I. W., Yoo S.-S., Panych L.P., Golby A., Mamata H., Maier S.S., Ratiu P., Charles R.G., Black P.M., Jolesz F.A., Kikinis R. (2003). Diffusion tensor and functional MRI fusion with anatomical MRI for image-guided neurosurgery. Medical Image Computing and Computer-Assisted Intervention-MICCAI 2003.

[bb0370] Tuch D.S., Reese T.G., Wiegell M.R., Makris N., Belliveau J.W., Wedeen V.J. (2002). High angular resolution diffusion imaging reveals intravoxel white matter fiber heterogeneity. Magn. Reson. Med..

[bb0375] Tuch D.S., Reese T.G., Wiegell M.R., Wedeen V.J. (2003). Diffusion MRI of complex neural architecture. Neuron.

[bb0380] Wan E.A., Van Der Merwe R. (2000). The unscented Kalman filter for nonlinear estimation. Adaptive Systems for Signal Processing, Communications, and Control Symposium 2000. AS-SPCC. The IEEE 2000. IEEE.

[bb0385] Weinstein D., Kindlmann G., Lundberg E. (1999). Tensorlines: Advection-diffusion based propagation through diffusion tensor fields. Proceedings of the Conference on Visualization’99: Celebrating Ten Years.

[bb0390] Westin C.-F., Maier S.E., Mamata H., Nabavi A., Jolesz F.A., Kikinis R. (2002). Processing and visualization for diffusion tensor MRI. Med. Image Anal..

[bb0395] Zhang H., Wang Y., Lu T., Qiu B., Tang Y., Ou S., Tie X., Sun C., Xu K., Wang Y. (2013). Differences between generalized q-sampling imaging and diffusion tensor imaging in the preoperative visualization of the nerve fiber tracts within peritumoral edema in brain. Neurosurgery.

